# Nonfatal Injuries 1 Week After Hurricane Sandy — New York City Metropolitan Area, October 2012

**Published:** 2014-10-24

**Authors:** Robert M. Brackbill, Kimberly Caramanica, Maret Maliniak, Steven D. Stellman, Monique A. Fairclough, Mark R. Farfel, Lennon Turner, Carey B. Maslow, Amanda J. Moy, David Wu, Shengchao Yu, Alice E. Welch, James E. Cone, Deborah J. Walker

**Affiliations:** 1 World Trade Center Health Registry, New York City Department of Health and Mental Hygiene, New York, New York; 2 School of Public Health, Emory University, Atlanta, Georgia; 3 Department of Epidemiology, Mailman School of Public Health, Columbia University, New York, New York

On October 29, 2012, Hurricane Sandy (Sandy) made landfall in densely populated areas of New York, New Jersey, and Connecticut. Flooding affected 51 square miles (132 square kilometers) of New York City (NYC) and resulted in 43 deaths, many caused by drowning in the home, along with numerous storm-related injuries (1). Thousands of those affected were survivors of the World Trade Center (WTC) disaster of September 11, 2001 (9/11) who had previously enrolled in the WTC Health Registry (Registry) cohort study. To assess Sandy-related injuries and associated risk factors among those who lived in Hurricane Sandy–flooded areas and elsewhere, the NYC Department of Health and Mental Hygiene surveyed 8,870 WTC survivors, who had provided physical and mental health updates 8 to 16 months before Sandy. Approximately 10% of the respondents in flooded areas reported injuries in the first week after Sandy; nearly 75% of those had more than one injury. Injuries occurred during evacuation and clean-up/repair of damaged or destroyed homes. Hurricane preparation and precautionary messages emphasizing potential for injury hazards during both evacuation and clean-up or repair of damaged residences might help mitigate the occurrence and severity of injury after a hurricane.

The Registry contains records of a cohort of 71,431 persons affected by events related to the WTC disaster in NYC, for which three waves of health data were collected during 2003–2012. Because Sandy occurred shortly after Wave 3 of data collection, which provided recent pre-hurricane health data, the Sandy survey was restricted to eligible enrollees who had completed a Wave 3 survey. The Sandy study sample included persons with a current address within an inundation zone in New York, New Jersey, or Connecticut (n = 4,435), as defined by the Federal Emergency Management Agency Modeling Task Force (2), and a comparison group of enrollees with addresses in the same states who resided outside the inundation zones (n = 4,435). Approximately 5 months after the storm (March 28, 2013), enrollees who met the selection criteria were contacted via e-mail and invited to participate in an Internet survey. Persons who did not complete the Internet survey (n = 6,353) were subsequently mailed paper questionnaires. Multiple mail and e-mail reminders were sent to nonrespondents, as well as three rounds of paper questionnaires. At the close of data collection (November 7, 2013), 4,558 surveys had been completed by 55.1% of enrollees in the inundation zones and 47.7% of enrollees not in an inundation zone (Figure).

The Sandy survey included questions on home evacuation, height of flood waters in home, degree of damage to home, activities related to storm response (e.g., rescue, clean-up, and repair), and a health assessment that included details of Sandy-related injury restricted to the first week after the hurricane to provide the respondent a distinct period for recall of the injury. Additional information was obtained about body part (e.g., arm/hand, leg, or foot), type of injury (e.g., cut, fracture, or strain), and whether medical care was received. The analysis was restricted to persons who provided complete injury information on the survey (n = 4,174).

In the NYC inundation zone alone, approximately 500 homes were destroyed, and 26,000 homes and businesses were registered for repairs (3). Of the 2,224 respondents who lived in an inundation zone, 42.1% reported home flooding, 48.9% evacuated from their home before, during, or after the storm, 19.2% had a home that was made uninhabitable or destroyed by the storm, and 10.4% sustained an injury in the first week after Sandy (Table 1). A much smaller proportion of the 1,950 respondents living in areas that were not inundated experienced Sandy-related exposures (i.e., 7.6% reported any flooding in the home, 13.8% evacuated, and 3.4% reported injuries).

Because of the elevated incidence of injuries among persons who resided in an inundation zone, analyses focused on those 231 injured persons. Over 70% (71.4%) reported two or more injuries (Table 2), representing 706 different injuries or an average of 3.1 injuries per injured person. The most common injury reported was arm/hand cut, followed by back strain/sprain and leg cut. Injuries were reported by 15.3% of men and 5.0% of women; the most common injury among men was arm/hand cut (n = 102), whereas among women it was foot strain (n = 23). Injury was also more commonly reported by persons aged 45–64 years (12.3%) and by those with household income of >$150,000 in 2010 (12.2%). Among the 231 injured persons, 25.1% reported they received treatment for their most serious injury at a hospital, emergency department (ED), or doctor’s office, although this differed by household income; 31.0% of injured persons with a household income of >$75,000 reported receiving treatment at a hospital, ED, or doctor’s office, compared with 17.5% among those with a household income of ≤$25,000. Persons might have been prevented from going to hospitals or other care facilities because of flooding, and those with higher income had the resources to go somewhere else (4).

Persons in inundation zones who reported flooding in their homes were more likely to report being injured, with likelihood of injury increasing with depth of flood water in the home. Rates of injury were lowest among those who had no flooding in their home and did not evacuate (3.0%), and highest among those who reported ≥3 feet (≥91 cm) of flooding in their home, regardless of whether they evacuated (26.1%) or did not evacuate (25.3%). Over 35% of injured persons who evacuated before (40.0%) or after (35.7%) the storm received treatment at a hospital, ED, or doctor’s office for their most serious injury. In addition, 39.3% of those who reported evacuating by walking or swimming through water reported an injury, and nearly half (45.5%) of this group reported seeking treatment for their injury at a hospital, ED, or doctor’s office. However, less than 9% of those injured who did not evacuate and had ≥3 feet of water in their homes sought treatment at a hospital, ED, or doctor’s office (8.3%). Hand/arm injuries, cuts or lacerations of the lower extremities, and back strains were frequent among persons who evacuated and had ≥3 feet of water in their homes. Among those whose homes were damaged or destroyed, injuries were reported almost exclusively by those who engaged in clean-up/repair (166 persons with clean-up/repair related injuries versus 17 without).

## Discussion

Typically, reports on hurricane-related morbidity are based on *ad hoc* active surveillance systems set up because of damage to or loss of public health infrastructure (5–7). The incidence of injury among survey respondents residing in inundation zones (10.4%) was similar to the 9% incidence of injuries reported among a random sample of 91 residents of Rockaway Peninsula, an inundated area of NYC heavily affected by Sandy (8). The actual incidence of Sandy-related injuries was likely higher because reporting of injuries was limited to those sustained in the first week after the storm and recovery has been a long-term process. Although multiple injuries were very common among injured enrollees, previous reports on hurricane-related injuries did not assess multiple injuries, but focused on serious injuries reported by EDs or other locations set up for immediate treatment (7). Many persons with injuries likely were unable to seek immediate treatment, and the finding that 25.1% sought treatment for their most serious injury likely underestimates the actual need for injury treatment. This would be consistent with the fact that less than 9% of those who did not evacuate and had ≥3 feet of water in their home reported receiving treatment for their most serious injuries.

The findings in this report are subject to at least four limitations. First, the findings are based on self-reported data collected 5–12 months after the event. Second, the overall response rate was 51.4%, leaving open the possibility of nonresponse bias; however, respondents and nonrespondents did not differ by income or socioeconomic status, and thus, misrepresentation of socioeconomic groups was less likely. Third, the sample is limited to those who experienced the 9/11 disaster in the NYC metropolitan area and cannot be used to make inferences about other populations affected by Sandy. Nevertheless, the survey was sent to 4,435 Registry enrollees identified by residence in well demarcated inundation zones. Fourth, information about the source or immediate cause of reported injuries, such as being struck by an object or falling, was not obtained. This limits interpretation of findings associated with specific Sandy-related exposures, such as evacuating, having a flooded home, or doing home repairs. Despite these limitations, the Registry provided a unique opportunity to rapidly survey large numbers of persons exposed to Sandy’s devastation by using a previously assembled cohort. For future storms with similar profiles, framing of prevention messages can be developed from these findings (e.g., that home repair can be hazardous).

What is already known on this topic?

Hurricanes are known to cause physical injuries either directly, often as a result of strong winds causing persons to fall or be hit with blown objects, or indirectly, when persons return to affected areas to conduct clean-up and repair activities. The most common types of hurricane-related injuries are cuts to upper extremities and back strain.

What is added by this report?

The degree of flooding in the home or surrounding area was directly related to the occurrence of injury, with 39% of those who evacuated by walking through water or swimming being injured, and 25% of those whose homes were flooded with ≥3 feet of water, regardless of whether they did or did not evacuate. Additionally, the greatest number of injuries occurred among persons who had a damaged or destroyed home and attempted to do clean-up or repair work (n = 166).

What are the implications for public health practice?

After hurricanes, injuries, particularly multiple injuries, are common and underreported. Injury surveillance and early precautionary messages concerning evacuation and clean-up or repairs of damaged residences would further enhance public health response by helping mitigate the occurrence and severity of injury after a hurricane.

Similar to reports regarding earlier hurricanes (e.g., Hurricanes Andrew, Katrina, and Irene), most reported injuries occurred after Sandy had passed and were associated with clean-up and repair activities (6,7). The types of injuries observed after other storms, including lacerations of upper extremities and back strains, were also the most frequently reported in this study of Sandy (9); this analysis did not include carbon monoxide poisonings or electrocution injuries which often occur after storm disasters. The NYC Department of Health and Mental Hygiene issued guidance via a press release on November 13, 2012, that contained precautions concerning debris removal and repair of homes after Sandy, including injury prevention advice (10). The findings on injuries sustained during the first week post-hurricane suggest the need for dissemination of injury-prevention advisories as early as possible post-hurricane, as well as before future hurricanes, if possible.

## Figures and Tables

**FIGURE:**
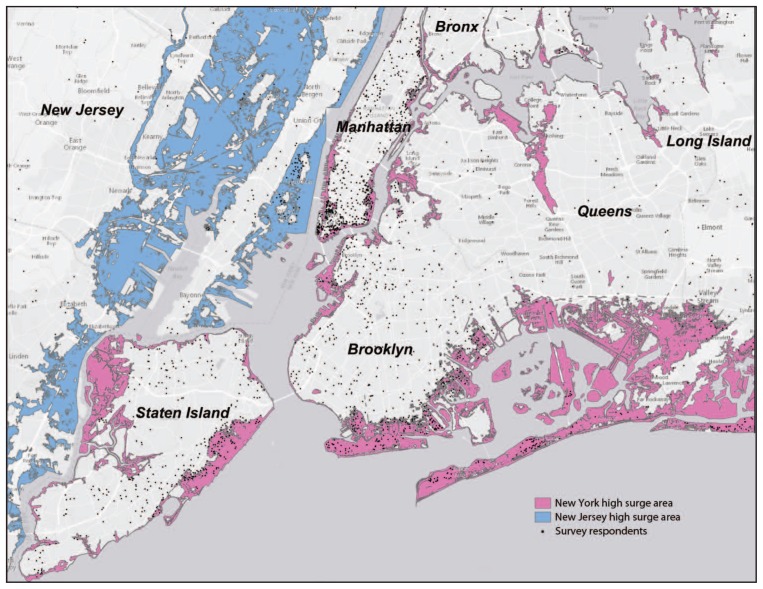
Hurricane Sandy inundation zones — New York City metropolitan area, October 2012^*^ **Source:** New York City Department of Health and Mental Hygiene, World Trade Center Health Registry. ^*^Map depicts 80% (n = 1,970) of respondents in the inundation zone sample and 47% (n = 991) of respondents in the sample of persons not in an inundation zone.

**TABLE 1:** Comparison of demographic and selected exposure characteristics of persons in inundations zones and not in inundation zones — Hurricane Sandy study, World Trade Center Health Registry, March 28–November 7, 2013

Characteristic	Inundation zone		Not in an inundation zone	
	
	No.*	(%)†	No.*	(%)†
**Overall**	**2,224**	**(100.0)**	**1,950**	**(100.0)**
**Sex**				
Male	1,164	(52.3)	1,174	(60.2)
Female	1,060	(47.7)	776	(39.8)
**Age on October 29, 2012 (yrs)**				
19–29	50	(2.2)	26	(1.3)
30–44	327	(14.7)	356	(18.3)
45–64	1,403	(63.1)	1,235	(63.3)
≥65	444	(20.0)	333	(17.1)
**Residence before Hurricane Sandy**				
New York City	1,782	(80.1)	1,056	(54.2)
Long Island	266	(12.0)	323	(16.6)
New Jersey	167	(7.5)	335	(17.2)
Other (e.g., New York state and Connecticut)	9	(0.4)	236	(12.1)
**Household income in 2010**				
≤$25,000	446	(20.1)	410	(21.0)
$25,001–$50,000	725	(32.6)	764	(39.2)
$50,001–$75,000	349	(15.7)	328	(16.8)
$75,001–$150,000	324	(14.6)	234	(12.0)
>$150,000	278	(12.5)	142	(7.3)
**Height of flood waters inside home**				
No flood water in home	1,238	(55.7)	1,770	(90.8)
<3 feet in living area or any flooding in nonliving area	602	(27.1)	137	(7.0)
≥3 feet	333	(15.0)	11	(0.6)
**Evacuated from home**				
Yes	1,087	(48.9)	270	(13.8)
No	1,127	(50.7)	1,664	(85.3)
**Degree of damage to home because of Hurricane Sandy**				
None or minimal damage	1,301	(58.5)	1,652	(84.7)
Damaged but habitable	455	(20.5)	231	(11.8)
Damaged and uninhabitable or destroyed	428	(19.2)	35	(1.8)
**Persons reporting injuries sustained in first week after Hurricane Sandy**				
Yes	231	(10.4)	67	(3.4)
No	1,993	(89.6)	1,883	(96.6)

* Includes sample with complete injury information.

† Denominator of percentages includes persons with missing data.

**TABLE 2:** Injuries sustained in the first week after Hurricane Sandy and treatments received among those who lived in an inundation zone, by demographic characteristics and selected exposures — Hurricane Sandy study, World Trade Center Health Registry, March 28–November 7, 2013

Characteristic	No. of respondents*	Injury			Treatment for most serious injury of those injured		Most common body area and type of injury (No. of reports)		
	
		No. of injured persons	% of persons with ≥1 injury	% of injured persons with >1 injury	% visiting hospital, emergency department, or doctor	% receiving other treatment			
**Total**	**2,224**	**231**	**10.4**	**71.4**	**25.1**	**16.9**	**Arm/Hand cut (116)**	**Back strain (113)**	**Leg cut (74)**
**Sex**									
Male	1,164	178	15.3	73.6	25.3	19.1	Arm/Hand cut (102)	Back strain (91)	Leg cut (62)
Female	1,060	53	5.0	64.2	24.5	9.4	Foot strain (23)	Back strain (22)	Leg strain (16)
**Age on October 29, 2012 (yrs)** †									
30–44	327	24	7.3	83.3	25.0	0.0	Arm/Hand cut (18)	Leg cut (14)	Back strain (10)
45–64	1,403	170	12.3	72.4	23.5	18.8	Arm/Hand cut (90)	Back strain (87)	Leg cut (53)
≥65	444	36	8.1	61.1	33.3	19.4	Back strain (16)	Foot strain (16)	Leg strain (11)
**Household income in 2010**									
≤$25,000	446	40	9.0	80.0	17.5	27.5	Arm/Hand cut (30)	Back strain (25)	Foot cut (12)
$25,001–$50,000	725	83	11.4	74.7	22.9	12.0	Back strain (45)	Arm/Hand cut (40)	Leg cut (31)
$50,001–$75,000	349	33	9.5	69.7	27.3	21.2	Back strain (16)	Arm/Hand cut (16)	Leg strain (12)
$75,001–$150,000	324	24	7.4	66.7	37.5	12.5	Arm/Hand cut (13)	Back strain (12)	Leg strain (9)
>$150,000	278	34	12.2	64.7	26.5	11.8	Foot strain (17)	Leg cut (10)	Arm/Hand cut (10)
**Did not evacuate home**									
No flooding in home	771	23	3.0	56.5	34.8	4.3	Back strain (14)	Leg strain (7)	Arm/Hand cut (6)
<3 feet in living area or any flooding in nonliving area	243	28	11.5	82.1	25.0	28.6	Arm/Hand cut (14)	Back strain (11)	Leg cut (9)
≥3 feet	95	24	25.3	79.2	8.3	25.0	Arm/Hand cut (16)	Back strain (13)	Foot cut (12)
**Evacuated home**									
No flooding in home	466	27	5.8	51.9	29.6	11.1	Back strain (11)	Leg strain (8)	Arm/Hand cut (7)
<3 feet in living area or any flooding in nonliving area	359	57	15.9	71.9	19.3	19.3	Back strain (26)	Arm/Hand cut (23)	Leg cut (16)
≥3 feet	238	62	26.1	83.9	30.6	16.1	Arm/Hand cut (41)	Leg cut (29)	Back strain (29)
**Among those who evacuated and had ≥3 feet of flooding when evacuated from flooded home (n = 238)**									
Before Sandy arrived	78	15	19.2	80.0	40.0	13.3	Leg cut (9)	Back strain (9)	Arm/Hand cut (8)
During the storm	62	19	30.6	78.9	26.3	21.1	Arm/Hand cut (12)	Leg cut (9)	Back strain (7)
After Sandy had hit	44	11	25.0	72.7	18.2	27.3	Arm/Hand cut (9)	Foot cut (6)	Foot strain (5)
After Sandy passed	45	14	31.1	100.0	35.7	7.1	Arm/Hand cut (9)	Back strain (6)	Body cut (5)
**How evacuated from flooded home**									
Walked, drove, rode not through water	131	28	21.4	85.7	21.4	17.9	Arm/Hand cut (17)	Back strain (13)	Leg cut (11)
Walked or swam through water	28	11	39.3	63.6	45.5	9.1	Arm/Hand cut (7)	Leg cut (6)	Back strain (4)
Drove or road through water, including in a boat	49	17	34.7	94.1	35.3	17.6	Arm/Hand cut (14)	Leg cut (9)	Back strain (9)
**Degree of damage to home and clean up/repair effort among those who lived in an inundation zone**									
No or minimal damage									
Clean up/Repair	217	14	6.5	64.3	21.4	7.1	Back strain (10)	Leg strain (5)	Arm/Hand cut (5)
No clean up/repair	1,084	26	2.4	38.5	26.9	11.5	Back strain (10)	Leg strain (7)	Foot strain (7)
Damaged but habitable									
Clean up/Repair	323	66	20.4	72.7	27.3	13.6	Arm/Hand cut (37)	Back strain (34)	Leg cut (21)
No clean up/repair	132	10	7.6	40.0	20.0	0.0	Foot strain (3)	Back strain (3)	Neck strain (2)
Damaged and uninhabitable or destroyed									
Clean up/Repair	349	100	28.7	85.0	24.0	22.0	Arm/Hand cut (67)	Back strain (50)	Leg cut (45)
No clean up/repair	79	7	8.9	57.1	14.3	42.9	Arm strain (3)	Foot strain (3)	Back strain (3)

* Restricted to persons with complete injury information.

† Because only one injury was reported among respondents aged 19–29 years, data for this age group were excluded.
